# NOVA1-Mediated SORBS2 Isoform Promotes Colorectal Cancer Migration by Activating the Notch Pathway

**DOI:** 10.3389/fcell.2021.673873

**Published:** 2021-10-08

**Authors:** Tao Zhang, Sixia Chen, Yi Peng, Changgang Wang, Xi Cheng, Ren Zhao, Kun Liu

**Affiliations:** ^1^Department of General Surgery, Ruijin Hospital, Shanghai Jiao Tong University School of Medicine, Shanghai, China; ^2^Shanghai Institute of Digestive Surgery, Ruijin Hospital, Shanghai Jiao Tong University School of Medicine, Shanghai, China; ^3^Tongji Hospital, Tongji University School of Medicine, Tongji University, Shanghai, China

**Keywords:** colorectal cancer, alternative splice, SORBS2-exon3, NOVA1, migration

## Abstract

**Background:** Gene expression and alternative splicing (AS) can promote cancer development *via* complex mechanisms. We aimed to identify and verify the hub AS events and splicing factors associated with the progression of colorectal cancer (CRC).

**Methods:** RNA-Seq data, clinical data, and AS events of 590 CRC samples were obtained from the TCGA and TCGASpliceSeq databases. Cox univariable and multivariable analyses, KEGG, and GO pathway analyses were performed to identify hub AS events and splicing factor/spliceosome genes, which were further validated in five CRCs.

**Results:** In this study, we first compared differentially expressed genes and gene AS events between normal and tumor tissues. Differentially expressed genes were different from genes with differentially expressed AS events. Prognostic analysis and co-expression network analysis of gene expression and gene AS events were conducted to screen five hub gene AS events involved in CRC progression: EPB41L2, CELF2, TMEM130, VCL, and SORBS2. Using qRT-PCR, we also verified that the gene AS events SORBS2 were downregulated in tumor tissue, and gene AS events EPB41L2, CELF2, TMEM130, and VCL were upregulated in tumor tissue. The genes whose mRNA levels were significantly related to the five hub gene AS events were significantly enriched in the GO term of cell division and Notch signaling pathway. Further coexpression of gene AS events and alternative splicing factor genes revealed NOVA1 as a crucial factor regulating the hub gene AS event expression in CRC. Through *in vitro* experiments, we found that NOVA1 inhibited gene AS event SORBS2, which induced the migration of CRC cells *via* the Notch pathway.

**Conclusion:** Integrated analysis of gene expression and gene AS events and further experiments revealed that NOVA1-mediated SORBS2 promoted the migration of CRC, indicating its potential as a therapeutic target.

## Introduction

Colorectal cancer is one of the most frequently diagnosed malignant cancers and the second leading cause of cancer-related deaths worldwide (Bray et al., 2018). Despite advances in diagnosis and treatment, the clinical outcomes for individual cases remain unsatisfactory (Laissue, 2019). Unfortunately, approximately 50% of CRC patients will develop recurrence after tumor resection, most of them within 2 years (Kindler and Shulman, 2001). Accumulated evidence in the past decades has indicated that recurrence and metastasis are complex and tightly regulated processes involved in numerous genetic alternations (Sveen et al., 2011). Therefore, exploring the biological mechanisms involved in CRC metastasis is vital in developing targeted therapies and improving prognosis of patients.

Although the total number of protein-coding genes in the human genome is nearly 20,000, the transcriptome is tremendously more complex (Xiong et al., 2018). A single RNA precursor can be spliced in different arrangements to synthesize distinct mRNA and protein variants with different structures and functions. This may be one of the main reasons for the increased complexity of biological processes (Climente-Gonzalez et al., 2017). The basic splicing patterns include alternate acceptor sites (AA), alternate donor sites (AD), exon skipping (ES), mutually exclusive exons (ME), alternate terminators (ATs), alternate promoters (APs), and retained introns (RI). Although exon array-based studies could help reveal the complexity of AS, the limitations of this technology are also apparent (Mojica and Hawthorn, 2010). With the rapid development of high-throughput technology, the functions of AS events can be widely and largely used in CRC. Using the Cancer Genome Atlas (TCGA) project data, Xiong et al. (2018) performed a systematic analysis to reveal critical AS events involved in CRC progression. Similar studies have also been performed in other types of solid tumors, such as lung cancer, esophageal carcinoma, and gastric cancer (Li et al., 2017; Lin et al., 2018; Mao et al., 2019). Specifically, the splicing factor SRSF6 directly binds to exon23 of ZO-1 mRNA to produce the aberrant ZO-1 isoform, which functions as a tumor-promoting gene in colorectal cancer (Wan et al., 2019). NOVA1, another splicing factor, promotes the inclusion of exons in the reverse transcriptase domain of hTERT to produce full-length hTERT transcripts, which accelerates non-small cell lung cancer growth (Ludlow et al., 2018).

Notch signaling is a primordial, evolutionarily conserved cell fate determination pathway involved in cancer biology, such as cancer stem cells, angiogenesis, and tumor immunity (Pannuti et al., 2010; Takebe et al., 2015). Activation of Notch signaling can induce cancer cell proliferation, invasion, and metastasis, such as colorectal cancer, gastric cancer, tongue cancer, and cervical cancer (Mu et al., 2017; Yue et al., 2018; Cui et al., 2019; Li et al., 2019; Zhang et al., 2019). Different gene isoforms regulate Notch signaling. Kent et al. (2011) presented evidence that the predominant TP63 isoform (ΔNp63α) promotes cellular quiescence *via* activation of Notch signaling. Pamarthy et al. (2016) reported that an isoform of the “a” subunit of V-ATPase (named as a2V-ATPase) regulated processing of Notch receptor and altered Notch signaling in breast cancer. QKI-5, an RNA-binding protein, regulates the alternative splicing of NUMB to suppress cell proliferation and prevent Notch pathway activation (Zong et al., 2014).

Due to the complex mutual regulation mediated by gene expression and AS events (Gabut et al., 2011; Pimentel et al., 2014), integrated analysis of gene expression and AS helps to better understand the mechanisms involved in cancer progression (Kalari et al., 2012; Bisognin et al., 2014; Feng et al., 2020a). In this study, we performed an integrated analysis of gene expression and gene AS events and identified critical gene AS events in CRC progression. Using GO analysis and a series of *in vitro* experiments, we further explored the potential mechanisms regulating gene AS events and AS events that induce CRC progression.

## Materials and Methods

### Data Extract

The transcriptome, AS events, and the prognostic data of 590 colorectal cancers were downloaded from the TCGA^1^ and TCGASpliceSeq^2^ databases, which included 50 colorectal cancers with matched normal tissue. Sixty-six genes of splicing factor genes were obtained from the SpliceAid 2 database^3^.

### Patients

Five consecutive, unselected CRC tissues and paired normal tissues were collected from surgical resections of CRC patients at Ruijin Hospital. None of the patients received adjuvant therapy before the operation. CRC tumors were histologically confirmed by two experienced pathologists independently. Written informed consent was obtained from all the patients with CRC. All aspects of this study were approved by the Research Ethics Committee of Shanghai Jiao Tong University. The UICC TNM classification system was used to determine the tumor stage. All tissue samples were stored in liquid nitrogen. The patient information is illustrated in Supplementary Table 2.

### Cell Lines, Cell Culture, and Transient Transfection

The CRC cell lines were purchased from the American Type Culture Collection and cultured in RPMI 1640 supplemented with 10% fetal bovine serum and 1% penicillin–streptomycin at 37°C with 5% CO_2_. Cancer cells were grown to 60–70% confluence, and the siRNAs were transfected using Lipofectamine 3000 according to the manufacturer’s protocol (Thermo Fisher Scientific, L3000015). After 48 h of transfer, RNA was collected, and then at 72 h, proteins were collected, and functional experiments were performed. The siRNA sequences used in this study were purchased from Bioegene (Shanghai) and are listed in Supplementary Table 3.

### Western Blot Analysis

The total protein of cancer cells was extracted using RIPA buffer supplemented with 1 mM phenylmethylsulfonyl fluoride. Protein concentration was measured using the BCA Protein Assay Kit (Cat. No. P0012S. Beyotime, China). Western blotting was performed according to our previous protocols (Feng et al., 2020b). The following primary antibodies were used: anti-GAPDH (ABCAM, ab8245, 1:1,000), anti-NOVA1 (SAB, 27594, 1:1,000), anti-NOTCH1 (CST, 3608S, 1:1,000), and anti-SORBS2 (SAB, 24643, 1:1,000). GAPDH was used as the internal control. Goat anti-mouse or goat anti-rabbit horseradish peroxidase-conjugated IgG was used as the secondary antibody (CST, 7076, 7074, 1:10,000). The membranes were incubated with secondary antibody for 2 h at room temperature, and bands were visualized using an enhanced chemiluminescence detection system (Amersham Bioscience, Piscataway, NJ, United States) according to the instructions of the manufacturer.

### Transwell Assays

After digestion with trypsin, 5 × 104 cells were seeded in the top chamber containing a polycarbonate membrane with 8-μm pores (Corning, Cambridge, MA, United States). The lower chamber was filled with 10% FBS as a chemoattractant. After 48 h, the cells of the inner membrane were scraped using a cotton swab, and the cells on the lower surface were fixed with 4% paraformaldehyde and stained with 0.1% crystal violet for counting.

### Quantitative Real-Time PCR and Percent Spliced-in Analysis

Total RNA from cancer cells and fresh tissues was extracted using TRIzol Reagent (Invitrogen) according to the protocols of the manufacturer. Then, the RNA molecules were dissolved in RNase-free DNase (TaKaRa, Dalian, China) and stored at −80°C. cDNA synthesis was performed using HiScript II Q Select RT SuperMix for qPCR (Vazyme, Nanjing, China). Finally, quantitative real-time PCR (qRT-PCR) was conducted using an Applied Biosystems 7500 Sequence Detection System (Applied Biosystems, Foster City, CA, United States) with AceQ^®^ Universal SYBR qPCR Master Mix (Vazyme, Nanjing, China). The sequences of the sense and antisense primers are listed in Supplementary Table 4. GAPDH was used as an internal control. Relative gene expression was calculated using the 2^–ΔΔCT^. According to the PSI analysis in the TCGASpliceSeq database, two paired primers were designed for quantitative analysis. One pair of primers was used to amplify the alternative exon (V1) and the other for the common exon (V2). The relative PSI value of every AS event in the CRC tissues was calculated as V1/V2.

### Coexpression Network Analysis

For these genes and AS events that are associated with poor or good prognosis, we introduced an algorithm called CoExpNetViz (version 2.0.2) (Tzfadia et al., 2015) in the Cytoscape software (Version 3.6.0, citation) to create a coexpression network. First, all the correlation between these genes were calculated, then the lower and upper percentile of the distribution of coexpression values are taken, and these genes were considered as coexpressed. Finally, these coexpressed that passed the cutoff were used to constructed a coexpression network.

### Gene Ontology Analysis

After we calculated and selected these differentially expressed genes (DEGs), we imported these DEGs into a database called Metascape^4^ (Zhou et al., 2019) for Gene Ontology enrichment analysis. Then we selected the “Express Analysis” button for default analysis mode. Terms with a *p*-value < 0.01, a minimum count of 3, and an enrichment factor >1.5 are collected and grouped into clusters based on their membership similarities. More specifically, *p*-values are calculated based on the accumulative hypergeometric distribution 2, and *q*-values are calculated using the Banjamini–Hochberg procedure to account for multiple testings.

### Hub Gene Detection

After the gene coexpression network was created, these gene pairs were then imported into CytoHubba, an algorithm used for identifying hub genes from complex interactomes (Chin et al., 2014). We ranked these genes with MCC score calculated from CytoHubba and selected the top 5 ones as the most important hub genes for downstream validation.

### Statistics Analysis

To compare the differentially expressed AS events between normal and tumor tissue, ANOVA analysis was performed using “stats” package in R. The differentially expressed genes between normal and tumor tissue were obtained using the “limma” package in R. Differentially expressed genes and AS events are displayed in a heatmap. Univariate Cox regression analysis was performed to obtain prognostic gene expression and gene AS events using the “survival” package in R and further displayed by forest plot. Other statistical analyses were performed using GraphPad Prism 6.0 (Inc., La Jolla, CA, United States). Comparisons between two groups were performed using a two-tailed unpaired Student’s *t*-test. Data are displayed as mean ± SD. Survival analysis was performed using the Kaplan–Meier method and compared using the log-rank test. Pearson’s correlation analysis was conducted to explore the correlation between the two variables. A value of *p* < 0.05 was considered statistically significant.

## Results

### Gene Expression Analysis and Gene Alternative Splicing Event Analysis Reveal Different Transcriptome Characteristics Involved in Colorectal Cancer Development

**FIGURE 1:**
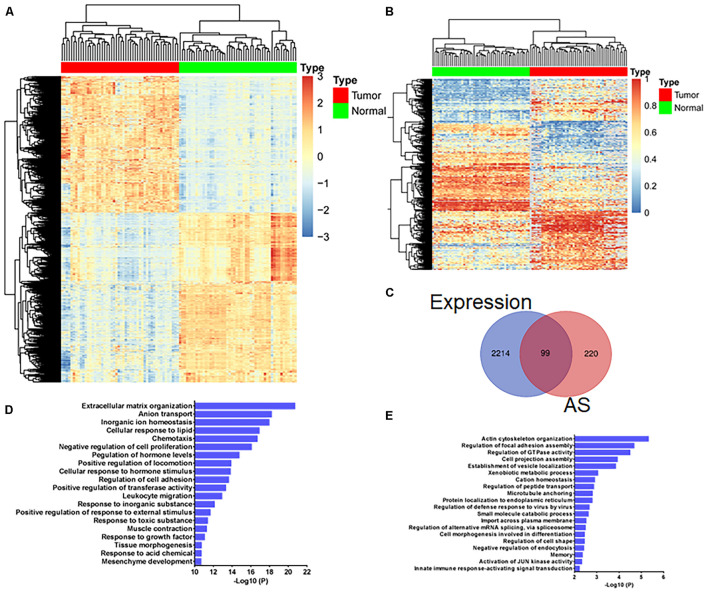
Gene expression analysis and gene alternative splicing (AS) event analysis reveal different transcriptome characteristics associated with colorectal cancer development. **(A)** Heatmap shows 2,313 differentially expressed genes (**A**, log2FC >1, adjusted *p*-value < 0.01) and **(B)** 338 differentially expressed AS events of 319 genes (difference >0.2, FDR <0.01) between 50 paired normal and tumor tissues. **(C)** Venn diagram displays the overlapping genes with differentially expressed mRNA levels and AS events between 50 paired normal and tumor tissue. **(D)** GO analysis of genes with differentially expressed mRNA levels and **(E)** genes with differentially expressed AS events.

By comparing differentially expressed genes between 50 paired normal and tumor tissues, 2,313 genes were obtained and displayed by heatmap (Log2FC >1, adjusted *p*-value < 0.01, Figure 1A and Supplementary Table 4). Through ANOVA analysis, we found 338 AS events of 319 genes that were differentially expressed between normal and tumor tissues (difference >0.2, FDR <0.01, Figure 1B and Supplementary Table 5). Using the Venn diagram, we found that most of the genes with differentially expressed mRNA levels were different from the genes with differentially expressed AS events (Figure 1C). GO analysis revealed that pathways enriched by genes with differentially expressed mRNA levels (Figure 1D) were also inconsistent with pathways enriched by genes with differentially expressed AS events (Figure 1E). These results indicate that the carcinogenic mechanisms involved in gene expression differed from gene AS events.

### Integrated Analysis Revealed Hub Gene Alternative Splicing Events in Colorectal Cancer

**FIGURE 2:**
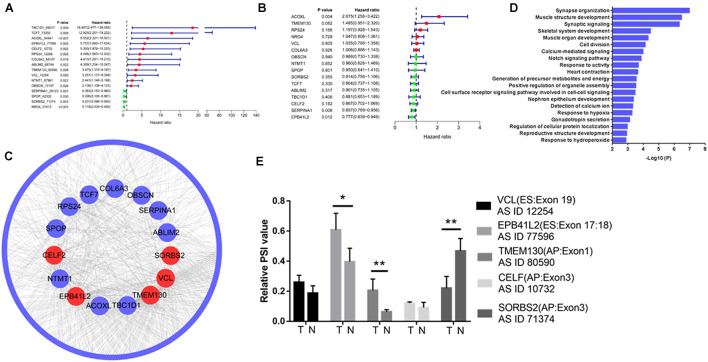
Integrated analysis reveals hub gene AS events in colorectal cancer. Differentially expressed AS events of genes **(A)** and differentially expressed genes **(B)** associated with prognosis in colorectal cancer. **(C)** Coexpression network of gene expression and gene AS events identified five hub gene AS events: EPB41L2, CELF2, TMEM130, VCL, and SORBS2 (*R* > 0.3, *p* < 0.001). **(D)** Gene Ontology (GO) analysis of the 300 genes whose mRNA levels were significantly related to the hub gene AS events in colorectal cancer (CRC). **(E)** EPB41L2, CELF2, TMEM130, VCL, and SORBS2 expression in five paired normal and tumor tissues by qRT-PCR. Each experiment was representative of three independent experiments. *p < 0.05, **p < 0.01, and ****p < 0.0001.

Of the 338 differentially expressed AS events, 12 upregulated AS events in tumor tissue were associated with poor prognosis, and four downregulated AS events in tumor tissue were associated with good prognosis (Figure 2A). However, of the 16 genes with prognostic AS events, the mRNA level of only one gene was related to poor prognosis (ACOXL, Figure 2B), and the mRNA level of only one gene correlated with good prognosis (EPB41L2, Figure 2B). By constructing a coexpression network of gene expression and gene AS events (*R* > 0.3, *p* < 0.001), we identified five hub gene AS events: EPB41L2, CELF2, TMEM130, VCL, and SORBS2 (Figure 2C and Supplementary Table 1). GO analysis revealed that the 300 genes whose mRNA levels were significantly related to the gene AS events were significantly involved in the GO terms (Figure 2D). We further examined the hub gene AS events and hub gene mRNA expression between five paired normal and tumor tissues by qRT-PCR, and found that the PSI of EPB41L2, TMEM130, and SORBS2 were different from tumor to normal (Figure 2E).

### Identification of Splicing Factor Regulating the Hub Gene Alternative Splicing Events

**FIGURE 3:**
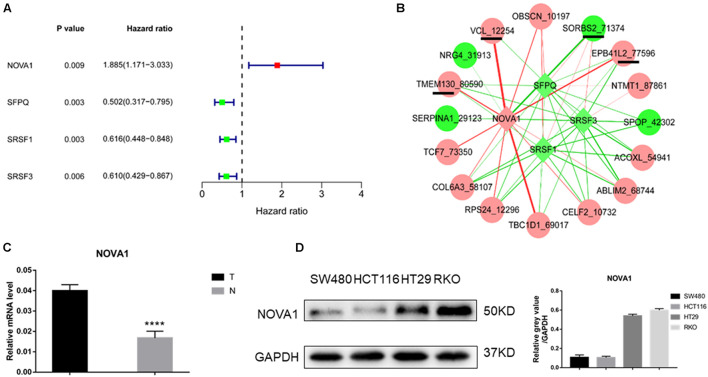
Identification of splicing factor regulating the hub gene AS events. **(A)** Of the 66 splice factor genes, three splicing factors were associated with good prognosis, and one splicing factor was related to poor prognosis by univariate Cox regression analysis. **(B)** Coexpression network of splicing factor gene expression, and gene AS events showed that four of the above five hub gene AS events were significantly related to NOVA1 mRNA expression. **(C)** qPCR was used to detect the expression of NOVA1 in five paired normal and tumor tissues. **(D)** The expression of NOVA1 in four CRC cell lines. Each experiment was representative of three independent experiments. *p < 0.05, **p < 0.01, ****p < 0.0001.

Univariate Cox regression analysis revealed that three splicing factors were associated with good prognosis, and one splicing factor was related to poor prognosis (Figure 3A). By constructing a coexpression network of splicing factor gene expression and gene AS events (*p* < 0.05), four of the above five hub gene AS events were significantly related to NOVA1 mRNA expression (Figure 3B). Then we detected the expression of NOVA1 by qRT-PCR, and the results showed that the tumor was higher than normal (Figure 3C). We examined the expression of NOVA1 and selected CRC cell lines with a high expression (RKO) of NOVA1 for further experiments (Figure 3D).

### NOVA1-Mediated SORBS2 Alter Splicing Promotes Colorectal Cancer Cell Migration *via* Notch Signaling Pathway

**FIGURE 4:**
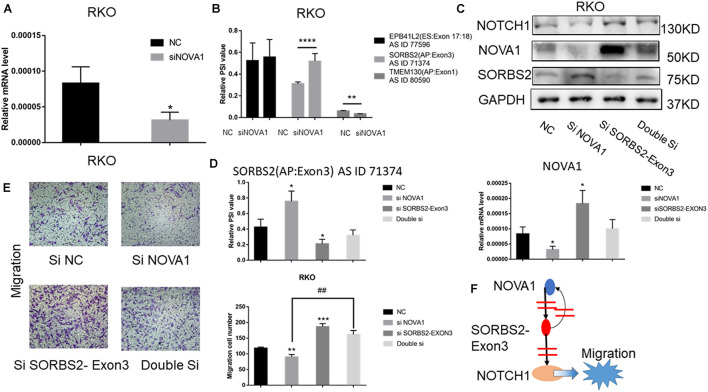
The signal axis NOVA1/SORBS2/NOTCH1 regulates the migration of CRC. **(A)** The percent spliced-in (PSI) value of EPB41L2, TMEM130, and SORBS2 in the NC group and the siNOVA1 group. **(B)** qPCR was used to detect the expression of NOVA1 in the NC group and the siNOVA1 group. **(C)** RKO was transfected with the indicated si-RNA. After that, Western blot was used to detect the expression of NOVA1, NOTCH1, SORBS2, and GAPDH. **(D)** qPCR was used to calculate the PSI VALUE and NOVA1 of each group. **(E)** Transwell experiment showed the migration ability of each group. **(F)** NOVA1 inhibits the production of SORBS2 containing exon3 through variable splicing. SORBS2 containing exon3 not only reversely inhibits NOVA1 but also inhibits NOTCH1, thereby promoting the migration of CRC. Each experiment was representative of three independent experiments. “*” represents comparing with the control. *p < 0.05, **p < 0.01, ***p < 0.001, and ****p < 0.0001, ##*p* < 0.01.

The above results indicated that the alternative splice of SORBS2-exon3 was downregulated in CRC tissue, which was defined as SORBS2-exon3. To explore the function of SORBS2-exon3 in CRC, after knocking down NOVA1 in CRC cells (Figure 4A), we also detected decreased AS events in TMEM130 and increased SORBS2 (Figure 4B). Of the four hub gene AS events, we found that SORBS2 was most apparently changed by NOVA1 knockdown. To verify the relationship between NOVA1, SORBS2-exon3, and NOVA1, RKO cells were transfected with siNOVA1 and/or siSORBS2-exon3. Western blotting showed that after knocking down NOVA1, SORBS2 containing exon3 increased, and the expression of NOTCH1 was suppressed. After knocking down SORBS2 containing exon3, the expression of NOTCH1 was increased. It is also surprising that the expression of NOVA1 was increased, indicating a negative feedback between NOVA1 and SORBS2-exon3, which may be the reason for CRC progression (Figure 4C). The qPCR results were consistent with the results of Western blotting (Figure 4D). Transwell experiments were preformed to detect the migration of CRC, and the result showed that NOVA1 knockdown-mediated CRC cell migration inhibition could be reversed by SORBS2-exon3 downregulation (Figure 4E), which indicated that NOVA1/SORBS2-exon3/NOTCH1 was involved in the migration of CRC (Figure 4F).

## Discussion

Colorectal cancer is a major threat to the lives of individuals worldwide, and the mechanisms involved in oncogenesis and cancer development are very complex (Tang et al., 2019). Gene alternative splicing makes the mechanisms more implicated because different isoforms of a gene may function in diverse ways. The inconsistency of different gene isoforms reminds us that we need to consider gene isoforms when studying genes. Using bioinformatics analysis, we first identified five hub gene AS events involved in CRC progression. We also verified that the gene AS events SORBS2 were upregulated in normal tissue, and gene AS events EPB41L2, CELF2, TMEM130, and VCL were downregulated in normal tissue by qRT-PCR. NOVA1 was identified as a critical splicing factor that regulates SORBS2 expression in CRC. Finally, *in vitro* experiments showed that NOVA1/SORBS2-exon3 promoted CRC cell migration through the Notch pathway. Therefore, a therapeutic regimen targeting SORBS2 may be a promising strategy to overcome CRC progression.

A previous analysis was performed to reveal the critical AS events associated with CRC development, such as AT in CXCL12, RI in CSTF3, AP in LBH, and ES in ALDH4A1 (Liu et al., 2018; Xiong et al., 2018). However, these studies did not simultaneously consider gene expression. Integrated analysis of gene expression and AS events can provide additional insights into the mechanisms of cancer progression (Koch, 2017; Song et al., 2019). By comparing differentially expressed genes and gene AS events, we found that the genes whose mRNA levels were significantly changed were different from those whose AS events were significantly changed. GO analysis revealed that the enriched GO terms using the differentially expressed genes also differed from the enriched GO terms using the genes with differentially expressed AS events. Coexpression network analysis of gene AS events and gene expression revealed five hub gene AS events involved in CRC progression: ES in EPB41L2, AP in CELF2, AP in TMEM130, ES in VCL, and AP in SORBS2. Overexpression of EPB41L2 in ovarian cancer has been reported to be associated with chemotherapy resistance (Menyhart et al., 2019). CELF2 suppression promotes gastric cancer cell proliferation and migration (Wang et al., 2018). Loss of vinculin can promote CRC metastasis and predict poor prognosis in patients with CRC (Lin et al., 2014). In ovarian cancer, SORBS2 suppresses metastatic colonization of cancer cells by eliciting a tumor-suppressive immune microenvironment (Zhao et al., 2018).

GO analysis indicated that the hub gene AS events and NOVA1 were associated with Notch pathway activation. The splice factor and splice factor kinase antiapoptotic isoform CLK1 can be induced by hypoxia to produce the antiapoptotic isoform caspase 9b in prostate cancer cells, which may be involved in antitumor therapy resistance (Bowler et al., 2018). Our results showed that NOVA1 was negatively correlated with SORBS2-exon3, and NOVA1 was positively correlated with NOTCH1 expression. After knocking down NOVA1, the migration cell number of RKO cells was less than that of NC. After knocking down SORBS2-exon3, the migration cell number of RKO cells was greater than that of NC. Furthermore, NOVA1 knockdown-mediated CRC cell migration inhibition could be reversed by SORBS2-exon3 downregulation. As we know, including mutations in KRAS, NRAS, PI3K, and BRAF genes are considered to be the key factors in the transformation of CRC (Vakiani et al., 2012; Hampel et al., 2018). In addition, increasing number of scholars are paying more attention to the impact of epigenetics, including altered splicing, on tumor progression (He et al., 2016; Amirkhah et al., 2019; Chen et al., 2020; Wang et al., 2020). Our data showed that after knocking down SORBS2-exon3, even if NOVA1 was knocked down together, the loss of the CRC migration function mediated by NOVA1 would be rescued. Remarkably, SORBS2-exon3 will reverse the expression of NOVA1. More attention should be paid to the altered splicing of SORBS2 in clinical treatment, which may be helpful for the treatment of CRC metastasis. We will further verify this in future experiments.

In conclusion, we revealed that gene expression-mediated CRC development mechanisms differ from the gene AS events induced by CRC progression. Further screening and validation confirmed that NOVA1 mediated SORBS2 alternative splicing induced CRC cell migration *via* the Notch pathway. Therefore, our results reveal a promising therapeutic strategy targeting SORBS2-exon3 in CRC.

## Data Availability Statement

The datasets presented in this study can be found in online repositories. The names of the repository/repositories and accession number(s) can be found in the article/Supplementary Material.

## Ethics Statement

The studies involving human participants were reviewed and approved by Ethics Committee of Shanghai Ruijin Hospital affiliated to Shanghai Jiao Tong University School of Medicine. The patients/participants provided their written informed consent to participate in this study.

## Author Contributions

RZ and KL were responsible for the conceptualizing and designing of the study. TZ, SC, and YP analyzed and interpreted the patient data and were major contributors in the writing of the manuscript. TZ, CW, XC, and KL collected and assembled the data. YP and SC performed the experiments. All authors read and approved the final manuscript.

## Conflict of Interest

The authors declare that the research was conducted in the absence of any commercial or financial relationships that could be construed as a potential conflict of interest.

## Publisher’s Note

All claims expressed in this article are solely those of the authors and do not necessarily represent those of their affiliated organizations, or those of the publisher, the editors and the reviewers. Any product that may be evaluated in this article, or claim that may be made by its manufacturer, is not guaranteed or endorsed by the publisher.

## Footnotes

1
https://tcga-data.nci.nih.gov/tcga/

2
http://projects.insilico.us.com/TCGASpliceSeq/index.jsp

3
http://193.206.120.249/splicing_tissue.html

4
http://metascape.org/gp/index.html
