# Supplementary figures and images for: NOVA1-Mediated SORBS2 Isoform Promotes Colorectal Cancer Migration by Activating the Notch Pathway
Source: Front Cell Dev Biol. 2021 Oct 8;9:673873. doi: 10.3389/fcell.2021.673873 (PMC8531477; doi:10.3389/fcell.2021.673873)

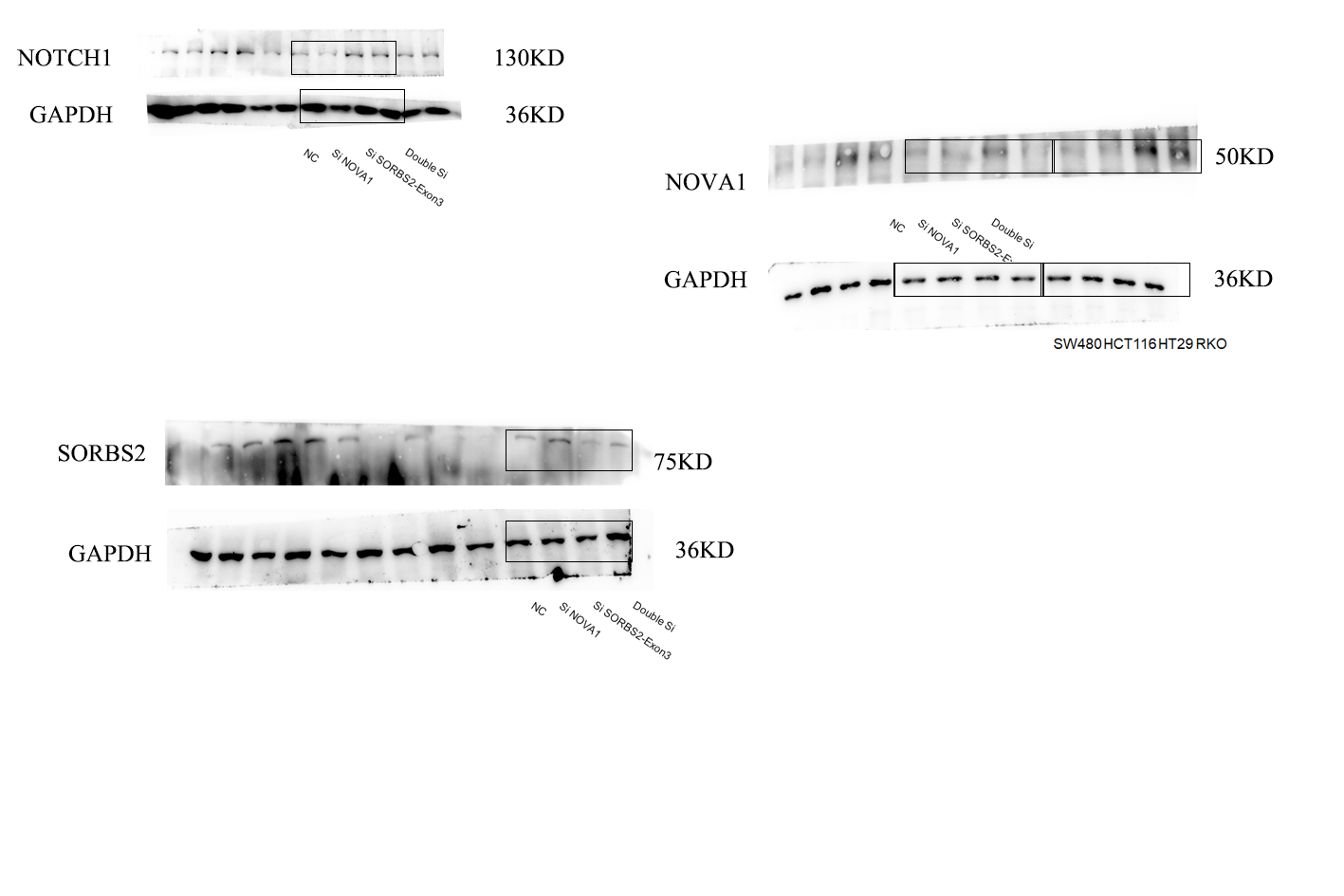

Supplement: Supplementary file 1 [file Image_1.TIF]
