# Supplementary material for: NOVA1-Mediated SORBS2 Isoform Promotes Colorectal Cancer Migration by Activating the Notch Pathway
Source: Front Cell Dev Biol. 2021 Oct 8;9:673873. doi: 10.3389/fcell.2021.673873 (PMC8531477; doi:10.3389/fcell.2021.673873)
Supplement: Supplementary file 2 [file Table_1.DOCX]

Table 1. The gene AS events associated with prognosis in CRC.

| AS ID | Gene name | Gene ID | Splice_type | Exons | From_exon | To_exon |
| --- | --- | --- | --- | --- | --- | --- |
| 69017 | TBC1D1 | 23216 | ES | 13:14 | 12 | 15 |
| 73350 | TCF7 | 6932 | ES | 6 | 5 | 7 |
| 54941 | ACOXL | 55289 | AT | 21 | NA | NA |
| 77596 | EPB41L2 | 2037 | ES | 17:18 | 14 | 20.1 |
| 10732 | CELF2 | 10659 | AP | 3 | NA | NA |
| 12296 | RPS24 | 6229 | ES | 5.2 | 4 | 6 |
| 58107 | COL6A3 | 1293 | ES | 3 | 2 | 4 |
| 68744 | ABLIM2 | 84448 | AT | 25 | NA | NA |
| 80590 | TMEM130 | 222865 | AP | 1 | NA | NA |
| 12254 | VCL | 7414 | ES | 19 | 18.2 | 20 |
| 87861 | NTMT1 | 28989 | AP | 2 | NA | NA |
| 10197 | OBSCN | 84033 | AT | 92 | NA | NA |
| 29123 | SERPINA1 | 5265 | AA | 2.1:2.2:2.3 | 1.1 | 2.4 |
| 42302 | SPOP | 8405 | AP | 1 | NA | NA |
| 71374 | SORBS2 | 8470 | AP | 3 | NA | NA |
| 31913 | NRG4 | 145957 | AT | 17.3 | NA | NA |
