# Supplementary material for: NOVA1-Mediated SORBS2 Isoform Promotes Colorectal Cancer Migration by Activating the Notch Pathway
Source: Front Cell Dev Biol. 2021 Oct 8;9:673873. doi: 10.3389/fcell.2021.673873 (PMC8531477; doi:10.3389/fcell.2021.673873)
Supplement: Supplementary file 4 [file Table_3.DOCX]

si-NOVA1：siRNA1：CAGACCACCGTTAATCCAGAT

siRNA2：ACCAAGTCCTCTCCATCTGAT

**siRNA3：CGAGTGTGCTTGATCCAGGGA(choose)**

si-SORBS2-Exon3：siRNA1：GCAATGGGCATGTTAGTATAC

siRNA2：GCAAGCTTTCCCTGCAATATT

**siRNA3：**GGTTTACCTGCTTCCTGTTAA **(choose)**
